# Predicting COVID-19 disease progression and patient outcomes based on temporal deep learning

**DOI:** 10.1186/s12911-020-01359-9

**Published:** 2021-02-08

**Authors:** Chenxi Sun, Shenda Hong, Moxian Song, Hongyan Li, Zhenjie Wang

**Affiliations:** 1 School of Electronics Engineering and Computer Science, Peking University, Beijing, People’s Republic of China; 2 Key Laboratory of Machine Perception (Ministry of Education), Peking University, Beijing, People’s Republic of China; 3 National Institute of Health Data Science, Peking University, Beijing, People’s Republic of China; 4 Institute of Medical Technology, Health Science Center of Peking University, Beijing, People’s Republic of China; 5 Institute of Population Research, Peking University, No.5 Yiheyuan Road, Beijing, 100871 People’s Republic of China

**Keywords:** COVID-19, Disease progression, Outcome early prediction, Irregularly sampled time series, Time-aware long short-term memory

## Abstract

**Background:**

The coronavirus disease 2019 (COVID-19) pandemic has caused health concerns worldwide since December 2019. From the beginning of infection, patients will progress through different symptom stages, such as fever, dyspnea or even death. Identifying disease progression and predicting patient outcome at an early stage helps target treatment and resource allocation. However, there is no clear COVID-19 stage definition, and few studies have addressed characterizing COVID-19 progression, making the need for this study evident.

**Methods:**

We proposed a temporal deep learning method, based on a time-aware long short-term memory (T-LSTM) neural network and used an online open dataset, including blood samples of 485 patients from Wuhan, China, to train the model. Our method can grasp the dynamic relations in irregularly sampled time series, which is ignored by existing works. Specifically, our method predicted the outcome of COVID-19 patients by considering both the biomarkers and the irregular time intervals. Then, we used the patient representations, extracted from T-LSTM units, to subtype the patient stages and describe the disease progression of COVID-19.

**Results:**

Using our method, the accuracy of the outcome of prediction results was more than 90% at 12 days and 98, 95 and 93% at 3, 6, and 9 days, respectively. Most importantly, we found 4 stages of COVID-19 progression with different patient statuses and mortality risks. We ranked 40 biomarkers related to disease and gave the reference values of them for each stage. Top 5 is Lymph, LDH, hs-CRP, Indirect Bilirubin, Creatinine. Besides, we have found 3 complications - myocardial injury, liver function injury and renal function injury. Predicting which of the 4 stages the patient is currently in can help doctors better assess and cure the patient.

**Conclusions:**

To combat the COVID-19 epidemic, this paper aims to help clinicians better assess and treat infected patients, provide relevant researchers with potential disease progression patterns, and enable more effective use of medical resources. Our method predicted patient outcomes with high accuracy and identified a four-stage disease progression. We hope that the obtained results and patterns will aid in fighting the disease.

## Background

Coronavirus disease 2019 (COVID-19) outbreaks have caused health concerns worldwide since December 2019; the disease was declared a pandemic by the World Health Organization (WHO) on 11 March 2020 [1]. Over seven million cases of COVID-19 have been reported worldwide, including more than 400,000 deaths (as of 15 June 2020) [2]. Even though the disease has been controlled in certain countries, the WHO director warns the pandemic is still ‘Speeding Up’ [3]. Because of its sudden onset, many hospitals are still facing medical resource shortages. For example, news in [4] reported a lack of medical resources in New Delhi. In [5], Arizona has experienced record-high hospital capacity as coronavirus cases climb. A reasonable allocation of resources according to patient condition is needed.

The solution to this problem involves determining the stages of disease progression by subtyping and predicting the outcome of COVID-19 patients. Then, targeted treatment and medical resource allocation can be carried out for patients in different stages. Recent studies [6–11] have used statistical methods to analyze COVID-19 progress by inpatient symptoms. However, different statistical results were obtained by considering different patient groups and different symptoms. At present, there is no clear division of the stages of COVID-19 progression.

Longitudinal disease analysis is the key to understanding disease progression, designing prognoses and developing early diagnostic tools. The time dynamics of disease can provide more information than static symptom observation [12]. Considering the complex patient states, the amount of interventions and the real-time requirement, the data-driven machine learning approaches by learning from electronic health records are the desiderata to help clinicians [13].

Many existing works have used machine learning methods for COVID-19 prediction tasks. We have summarized them in Table 1. For example, in most method of [27] and in [1, 14–19], authors used non-deep learning methods, such as k-NN, LR, Cox, SVM and DT to classify CT/X-ray images and predict the outcomes of COVID-19 patients. However, in terms of prediction accuracy, non-deep learning is not as good as deep learning methods. Deep learning methods can train the parameters with complex nonlinearity to learn the data structures and have achieved state-of-the-art in many medical prediction tasks [28–30]. Thus, many current works apply deep learning methods for COVID-19 prediction tasks [17, 19–26]. However, these methods either use the simple multi-layer perceptron for predicting or use the convolutional structures for image classification. Both the above methods ignored the temporal development of patient’s status. In the real-world patient records, except for the basic information, vital signs, test values and diagnoses are both time series, especially for the blood samples of COVID-19 patients, the data we used in this paper.

**Table 1:** The conclusion of machine learning methods used in COVID-19 prediction tasks

Non-deep learning methods				Deep learning methods		
Statistics	Regression	SVM	Decision tree	Basic NN	CNNs	RNNs
LDA [14] NB [15] k-NN [16]	LR [17, 18] Cox [19]	[17]	RF [17] XGBoost [1]	BPNN [17, 19, 20], GRNN [21], RBFNN [21], PNN [21]	Basic CNN [22], Transfer CNN [23], GDCNN [24], COVID-Net [25], COVNet [26]	/

We use the abbreviations of methods and the full names are listed in Table 1

Recently, a deep learning method, recurrent neural network (RNN) [31] can efficiently model temporal sequences. It uses recursion in the direction of sequence evolution to learning the relations among past, present and future. But the basic RNN has the long-term dependency problems [32]. Meanwhile, RNN only process uniformly distributed longitudinal data while COVID-19 patient blood samples are distributed nonuniformly with irregular time intervals between observations. Thus, a method that can model this irregular time series of COVID-19 patients is needed.

In this paper, we retrospectively analyzed the blood samples of 485 patients from the region of Wuhan, China. The medical records collected with standard case report forms, including epidemiological, demographic, clinical, laboratory and mortality outcome information, from an online open dataset under an MIT license. We applied a temporal deep learning method Time-aware Long Short-term Unit (T-LSTM) to model the irregular time series of COVID-19 patients. T-LSTM can predict the mortality with more than 98% accuracy before 3 days. Meanwhile, we have discovered four stages of COVID-19 patients. According to the different stages, we gave the analysis of the patient’s state and found the related biomarkers and complications.

## Methods

In this section, we first introduce the COVID-19 dataset and the data preprocessing process. Then, we describe the methods for mortality prediction and disease progression in detail.

### Dataset description

**Fig. 1:**
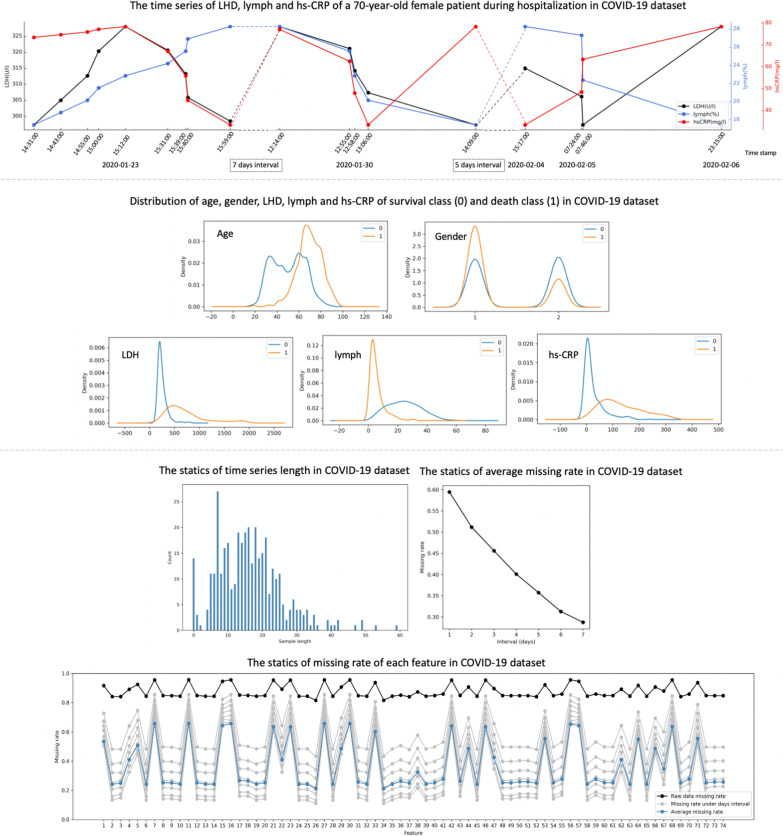
Examples and statistics of COVID-19 dataset. The first block is a line chart of an example in dataset - a 70-year-old female patient. It draws the time series of LHD, lymph and hs-CRP during hospitalization; The second block is the distributions of age, gender, LHD, lymph and hs-CRP of survival class (0) and death class (1); The third block is the statistics about dataset. It contains the counts of time series length, the statistics of overall missing rate and the statistics of each feature’s missing rate under different sampling rate

Blood index values can reflect a COVID-19 patient’s physical condition [10]. COVID-19 patients’ blood samples were collected between 10 January and 18 February 2020 at Tongji Hospital of Tongji Medical College, Huazhong University of Science and Technology, Wuhan, China [33]. The dataset contains 80 characteristics from 375 patients with 6120 records as a training set and 110 patients with 757 records as a test set. A case of sample is shown in Fig. 1. It draws lines of the time series of LHD, lymph and hs-CRP of a 70-year-old female patient during hospitalization. We can see the time intervals between two observations are irregular, which could be a few minutes or even days.

The detailed statistical information of demographic and 74 clinical laboratory test features is listed Table 2. For example, in the dataset, the average age of patients is 58.83, the survival rate is 53.6% and the ratio of male to female is about 1.5:1. We also list the range and mean value of each feature. In Fig. 1, we display the distributions of some features (age, gender, LHD, lymph and hs-CRP) of survival class (0) and death class (1).

**Table 2:** Demographic, laboratory and outcome information of 375 samples in training dataset

Characteristics^a^	Statistics^b^	Characteristics^a^	Statistics^b^
Demographics		Outcomes	
Age, mean (years)	58.83	Survival, count and rate	201, 53.6%
Gender	Male 224; Female 151	Mortality, count and rate	174, 46.4%
Lab test mean (min, max) patient’s last measurements			
cTnI	747.76 (1.9, 43,905.19)	glucose	8.37 (2.43, 32.37)
Hemoglobin	124.89 (6.4, 178.0)	neutrophils count	7.03 (0.85, 31.43)
Serum chloride	102.73 (77.7, 138.2)	Direct bilirubin	9.5 (1.7, 216.3)
Prothrombin time	16.01 (11.69, 84.22)	Mean platelet volume	10.89 (9.04, 14.0)
procalcitonin	0.99 (0.02, 49.34)	ferritin	1634.37 (26.8, 42,402.91)
Eosinophils(%)	0.56 (0.0, 5.61)	RBCW-SD	41.78 (32.5, 83.3)
sIL-2R	961.52 (61.0, 5608.04)	Thrombin time	18.17 (13.0, 133.67)
Alkaline phosphatase	84.86 (37.18, 481.5)	Lymphocyte(%)	16.45 (0.6, 54.83)
albumin	33.06 (19.1, 45.07)	Anti-HCV	0.13 (0.03, 1.85)
basophil(%)	0.21 (0.0, 1.38)	D-D dimer	6.2 (0.21, 29.82)
Interleukin 10	12.5 (5.0, 500.0)	Total cholesterol	3.66 (0.66, 6.11)
Total bilirubin	16.28 (2.95, 276.0)	AST	56.53 (8.0, 1858.0)
Platelet count	187.78 (1.2, 472.5)	Uric acid	297.47 (57.0, 1001.0)
monocytes(%)	6.66 (0.62, 31.62)	HCO3-	22.59 (6.3, 29.7)
antithrombin	87.57 (20.0, 136.0)	calcium	2.1 (1.35, 2.5)
Interleukin 8	88.08 (5.0, 6385.85)	NT-proBNP	3166.26 (5.57, 70,000.0)
indirect bilirubin	6.85 (1.0, 79.3)	LDH	492.12 (143.0, 1867.0)
RDW	12.94 (10.91, 22.91)	platelet large cell ratio	31.63 (16.48, 54.1)
Neutrophils(%)	76.09 (15.9, 98.1)	Interleukin 6	130.37 (1.5, 5000.0)
Total protein	66.06 (36.7, 79.33)	FDP	44.29 (4.0, 182.4)
Anti-TP	0.17 (0.02, 8.74)	monocytes count	0.54 (0.08, 22.75)
Prothrombin activity	80.97 (16.3, 136.64)	PLT distribution width	12.97 (8.42, 25.3)
HBsAg	10.35 (0.0, 250.0)	globulin	32.96 (13.8, 46.04)
mean corpuscular volume	89.75 (62.3, 110.73)	γ-GT	51.79 (10.6, 555.25)
hematocrit	37.0 (19.9, 51.3)	INR	1.3 (0.86, 10.51)
WBC	15.18 (0.8, 1726.6)	basophil count(#)	0.02 (0.0, 0.12)
Tumor necrosis factorα	12.07 (4.0, 168.0)	2019-nCoV nucleic acid	−1.0 (−1.0, −1.0)
MCHC	344.17 (286.0, 464.33)	MCH	30.9 (20.8, 47.67)
fibrinogen	4.38 (0.5, 8.89)	APTT	41.72 (25.59, 100.27)
Interleukin 1β	6.56 (5.0, 79.44)	hs-CRP	72.49 (0.2, 320.0)
Urea	9.74 (2.17, 68.4)	anti-HIV	0.1 (0.05, 0.26)
lymphocyte count	1.06 (0.13, 35.53)	serum sodium	141.06 (122.8, 171.4)
PH value	6.47 (5.0, 7.54)	thrombocytocrit	0.21 (0.05, 0.49)
Red blood cell count	7.58 (0.1, 749.5)	ESR	32.33 (2.0, 102.0)
Eosinophil count	0.03 (0.0, 0.33)	GPT	42.6 (5.0, 1508.0)
Corrected calcium	2.34 (1.78, 2.67)	eGFR	81.07 (2.0, 164.7)
Serum potassium	4.45 (3.1, 9.86)	creatinine	120.38 (39.25, 1497.0)

^a^Characteristics have three types - demographics (age and gender), outcomes (survival and mortality) and laboratory test (74 items)

^b^ Statistics is statistical data for corresponding characteristics, such as rate, mean value and range. The statistical methods are described in each column

This COVID-19 blood test data is publicly available at https://github.com/HAIRLAB/Pre_Surv_COVID_19.

### Dataset preprocessing

First, we attempted to find a suitable time measurement granularity. In the raw dataset, the lengths of sequences are unequal and different sampling times result in missing data, with an 85% missing rate on average. The missing rate is expressed in Eq. 1. *N*_*missing*_ means the number of time points with missing data in one time series. *N*_*all*_ means the number of time points in that time series. The presence of vacancies has a large impact on data quality, resulting in unstable predictions and other unpredictable effects [34]. We used 3 days as the basic sampling interval, reducing the average *mr* below 30%. The time series length of raw data, the average missing rate and the missing rate for each feature are shown in Fig. 1.

1 $$mr=\frac{N_{missing}}{N_{all}}$$

Meanwhile, for feature selection, using all 74 laboratory test features is unrealistic. To address the high missing rate, repeated features and collection difficulties, we considered three key features: lactic dehydrogenase (LDH), lymphocytes (lymph) and high-sensitivity C-reactive protein (hs-CRP). These features contain specific research biomarkers of COVID-19 patients [33] and can be easily collected in any hospital. Considering that only three features may not achieve high prediction accuracy, we also select 40 features (listed in Table 7) with missing rate less than 30% for comparative experiment.

### T-LSTM

**Fig. 2:**
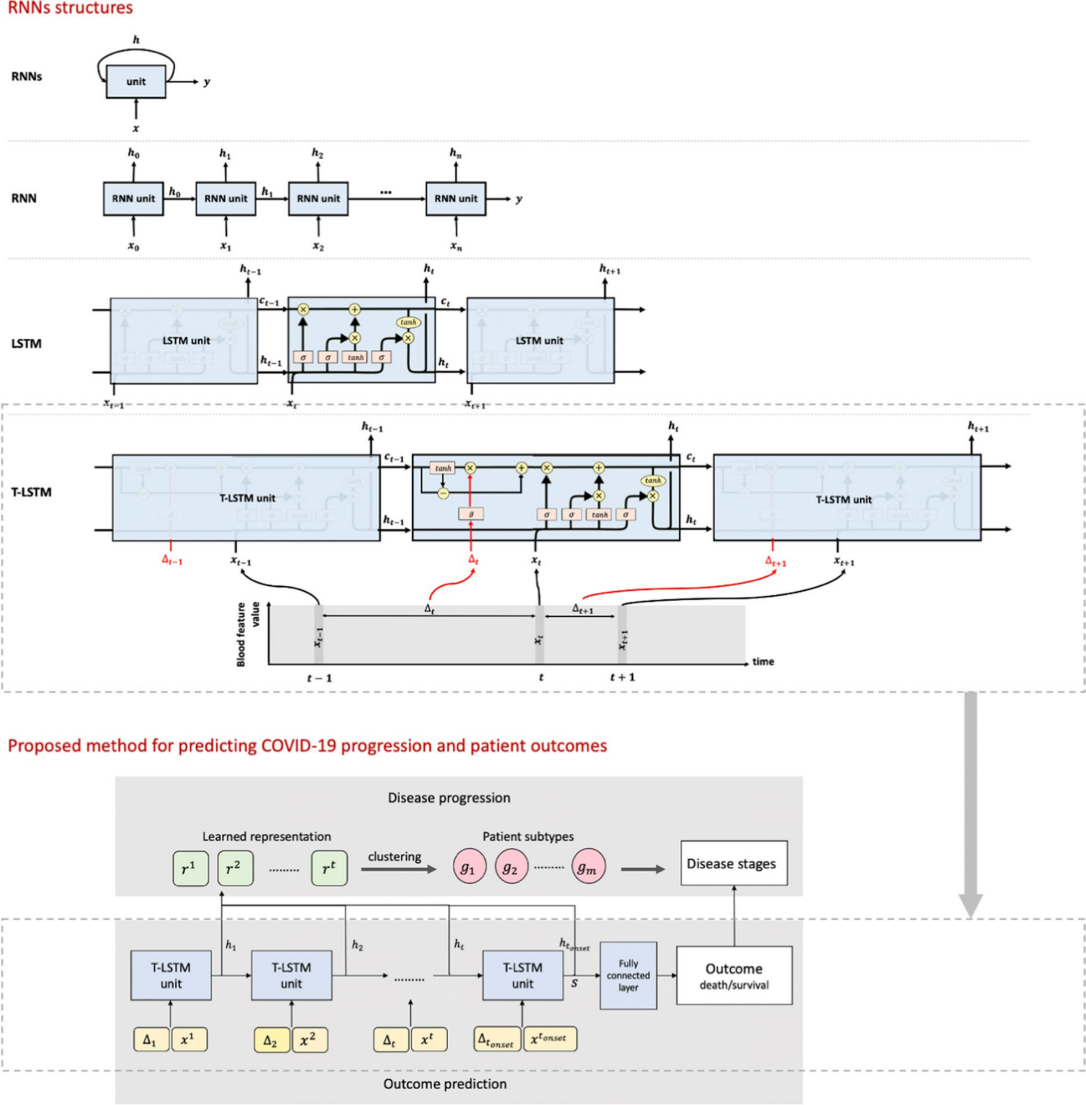
Structures of the methods. The first block shows the structure of RNNs, including basic RNN, LSTM and our T-LSTM; The second block shows the structure that how to use T-LSTM to complete the outcome prediction task (lower grey area) and disease progressing task (upper grey area)

Recurrent neural networks (RNNs) [31] (the first structure in Fig. 2) are deep network architectures designed to model temporal sequences. They take sequence data as input, recursion occurs in the direction of sequence evolution, and all units are chained together. In basic RNN (the second structure in Fig. 2), the current state *h*_*t*_ is affected by the previous state *h*_*t* − 1_ and the current input *x*_*t*_ and is described as *h*_*t*_ = *σ*(*Wx*_*t*_ + *Uh*_*t* − 1_ + *b*), where *σ* is an activation function, and *W*, *U* and *b* are learnable parameters. Long Short-Term Memory (LSTM) [32] (the third structure in Fig. 2) is a variant of RNN that is adept at solving long-term dependency problems. A standard LSTM unit consists of a forget gate *f*_*t*_, an input gate *i*_*t*_, memory cells *C*_*t*_, $\overset{\sim }{C_t}$ and an output gate *o*_*t*_.

However, RNNs only process uniformly distributed longitudinal data by assuming that the sequences have an equal distribution of time differences. COVID-19 patient blood samples are distributed nonuniformly. For example, the time gap between two sequential records could be hours or days. Time-aware Long Short-Term Memory (T-LSTM) [35] (the fourth structure in Fig. 2) incorporates the elapsed time information into LSTM. It applies a memory discount to capture the irregular temporal dynamics. T-LSTM can be formulated as:

2 $$\begin{array}{*{20}l} {C_{{t - 1}}^{S} = \tanh \left( {W_{d} C_{{t - 1}} + b_{d} } \right)} \hfill & {{\text{Short-term}}\;{\text{memory}}} \hfill \\ {\hat{C}_{{t - 1}}^{S} = C_{{t - 1}}^{S} * g\left( {\Delta _{t} } \right)} \hfill & {{\text{Discounted}}\;{\text{short-term}}\;{\text{memory}}} \hfill \\ {C_{{t - 1}}^{T} = C_{{t - 1}} - C_{{t - 1}}^{S} } \hfill & {{\text{Long-term}}\;{\text{memory}}} \hfill \\ {C_{{t - 1}}^{ * } = C_{{t - 1}}^{T} - \hat{C}_{{t - 1}}^{S} } \hfill & {{\text{Adjusted}}\;{\text{previous}}\;{\text{memory}}} \hfill \\ {f_{t} = \sigma \left( {W_{f} x_{t} + U_{f} h_{{t - 1}} + b_{f} } \right)} \hfill & {{\text{Forget}}\;gate} \hfill \\ {i_{t} = \sigma \left( {W_{i} x_{t} + U_{i} h_{{t - 1}} + b_{i} } \right)} \hfill & {{\text{Input}}\;{\text{gate}}} \hfill \\ {\mathop {C_{t} }\limits^{\sim } = \tanh \left( {W_{c} x_{t} + U_{c} h_{{t - 1}} + b_{o} } \right)} \hfill & {{\text{Candidate}}\;{\text{memory}}} \hfill \\ {C_{t} = f_{t} * C_{{t - 1}}^{ * } + i_{t} * \mathop {C_{t} }\limits^{\sim } } \hfill & {{\text{Current}}\;{\text{memory}}} \hfill \\ {o_{t} = \sigma \left( {W_{o} x_{t} + U_{o} h_{{t - 1}} + b_{o} } \right)} \hfill & {{\text{Output}}\;{\text{gate}}} \hfill \\ {h_{t} = o_{t} * \tanh \left( {C_{t} } \right)} \hfill & {{\text{Current}}\;{\text{hidden}}\;{\text{state}}} \hfill \\ \end{array}$$

In Eq. 2, based on the basic LSTM, T-LSTM possesses some new designs. ${C}_{t-1}^S$ component learns the short-term memory of sequence by learnable network parameters. ${C}_{t-1}^T$ is the long-term memory calculated from the former memory cell *C*_*t* − 1_ with getting rid of ${C}_{t-1}^S$. ${C}_{t-1}^S$ is adjusted to the discounted short-term memory ${\hat{C}}_{t-1}^S$ by the elapsed time function *g*(Δ_*t*_). The previous memory ${C}_{t-1}^{\ast }$ is changed to the complement subspace of ${C}_{t-1}^T$ combined with ${\hat{C}}_{t-1}^S$.

We use a log calculation for the elapsed time function. Δ_*t*_ describes the time gap between two records at two sequential time steps *t* and t − 1. *T*_*t*_ is the actual time at time step *t*.

3 $$g\left( {\Delta _{t} } \right) = \frac{1}{{\log \left( {e + \Delta _{t} } \right)}}, \quad\Delta _{t} = T_{t} - T_{{t - 1}}$$

### Analysis strategy

**Fig. 3:**
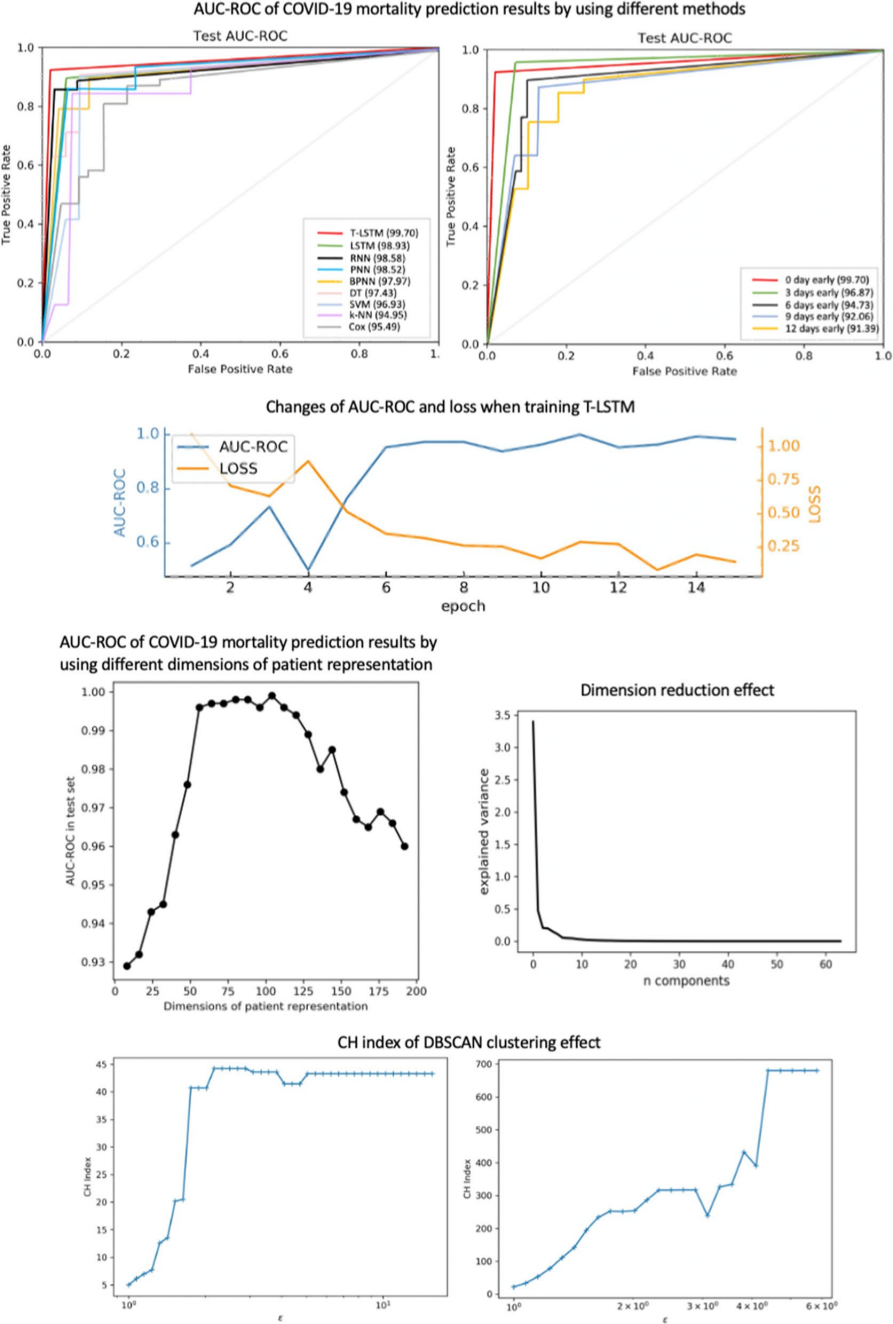
The results of outcome prediction. The first line’s charts are the AUC-ROC of mortality prediction results using baselines; The second line’s chart is the changes of accuracy and loss during training T-LSTM; The third line’s charts are the dimension experiments. They show the accuracy of mortality prediction by using different representation dimensions and the effect of representation dimension reduction; The fourth line’s charts are the effect when using DBSCAN

We first describe the two tasks in this study and then introduce the specific methods. The whole method process is shown in Fig. 3.

***Task 1 (Outcome prediction*** *) A set of labeled patient data is represented as* $\mathcal{D}=\left\{\left({x}_i,{c}_i\right)\in \left(X,C\right)|i=1,\dots, n\right\}$*. X is a time series set of patients, where* ${x}_i=\left\{{x}_i^t|t=1,\dots, {t}_{onset}\right\}$ *represents a patient’s records over t time steps; specifically,* ${x}_i^t$ *is multivariate data, and each dimension is a clinical record represented by a numeric vector. C* ∈ {0, 1} *is the outcome, where class* 0 *means death and class* 1 *means survival. The outcome prediction task aims to predict patient outcomes by the prediction function f* : *X* → *C*

***Task 2 (Temporal patient subtyping / Disease progression mining)*** *The goal is to find patient groups G* = {*g*_*i*_| *i* = 0, …, *m*} *with similar feature representation* $R=\left\{{r}_i^t|i=0,\dots, n;t=0,\dots, {t}_{onset}\right\}$. ${r}_i^t$ *is the representation of clinical record* ${x}_i^t$ *at time t. Then, the patient groups G distributed over time are used to analyze the stages of disease progression*

In COVID-19 patient outcome prediction task, T-LSTM is used to handle patient record sequences and then make the prediction. The process is displayed in the proposed method of Fig. 2, in the lower gray area.

For a patient *i*, the input of T-LSTM at time step *t* is a three-dimensional feature vector ${x}_i^t=\left[{v}_{LDH},{v}_{lymphocytes},{v}_{hs- CRP},\right]$ with time gap Δ_*t*_. The output is the state representation *s*_*i*_ at the last time step. We apply this outcome prediction task as a binary classification task, with two classes: death and survival.

The cross-entropy [36] is mainly used to measure the difference between two probability distributions. We expect our predicted distribution of patient outcomes to be closer to the true distribution. Thus, we use the cross-entropy loss function in Eq. 4. Besides, when using sigmoid active function, this loss can avoid the reduced learning rate causing by traditional mean square error loss when gradient decreases.

4 $$L={L}_{CE}\left(C,\hat{C}\right)=-{\sum}_xp(x) logq(x)=-{\sum}_{i=1}^n\hat{c_i}{\log}{c}_i+\left(1-\hat{c_i}\right){\log}\left(1-{c}_i\right)$$

*p*(*x*) is the prior probability (true label vector) and *q*(*x*) is the prediction probability (predicted results vector). Correspondingly, $\hat{C}$ is the real class of input data, and *C* represents the prediction class.

In COVID-19 patient disease progression task, temporal patient subtyping can uncover the dynamic characteristics of diseases by significantly nuanced subtyping, which leads to the potential stages of disease progression. We addressed the issue by building a time stage reference and providing a low-dimensional representation of each subject, encoding his or her position with respect to this reference.

The method structure is displayed in the upper gray area of proposed method in Fig. 2. It has 4 steps: 1) Acquisition of patient representation *r*^*t*^. We used the hidden state *h*_*t*_, extracted from every T-LSTM unit, as the patient’s representation *r*^*t*^ at time step *t*. 2) Dimension reduction of *r*^*t*^. For better demonstration, we used the t-distributed Stochastic Neighbor Embedding (t-SNE) [37] method to reduce these high-dimensional vectors *r*^*t*^ into two dimensions. 3) Obtaining the patient group set *G*. As prior information about the patient groups was not available, we acquired patient groups by applying an unsupervised clustering method, the Density-Based Spatial Clustering of Applications with Noise (DBSCAN) [38], on *r*^*t*^. 4) Analysis of *G* and stages of disease progression. The mortality rate *MR*, and the average time distance *TD* were calculated as the analysis criteria.

5 $$MR=\frac{N_{death}}{N_{patient}}$$

6 $$TD=\frac{1}{\mid {g}_k\mid }{\sum}_{x_i^t\in {g}_k}\left({T}_{t_{onset}}-{T}_t\right)$$

Equation 5 expresses the mortality rate. *N*_*death*_ is the number of patients with the death outcome and *N*_*patient*_ is the total number of patients. Eq. 6 expresses the average time distance. *T*_*t*_ means the current prediction time and ${T}_{t_{onset}}$ means the time of outcome. ∣*g*_*k*_∣ is the number of patients in group *g*_*k*_.

### Evaluation metrics

The prediction results were evaluated by assessing the area under the curve of the Receiver Operating Characteristic (AUC-ROC). The ROC is a curve of the True Positive Rate (TPR) and the False Positive Rate (FPR). TN, TP, FP and FN represent true positives, true negatives, false positives and false negatives, respectively.

7 $$TPR=\frac{TP}{TP+ FN}$$

8 $$FPR=\frac{FP}{TN+ FP}$$

The patient groups obtained by unsupervised clustering were evaluated by the Calinski-Harabaz Index (CH), which measures the covariance of data within a class and between classes. A larger CH value indicates a better clustering performance. In Eq. 9, *m* is the number of data and *k* is the number of groups. *B*_*k*_ and *W*_*k*_ respectively represent the covariance matrices between groups and within groups.

9 $$CH=\frac{tr(B_k)}{tr(W_k)}\frac{m-k}{k-1}$$

When we get the stages of COVID-19 patients, we used Kullback-Leibler Divergence (KL divergence) to analyze patient characteristics through each laboratory test feature. KL divergence can measure the degree of difference between two probability distributions. For each feature, we first establish the Gaussian distribution $\mathcal{N}\left(\mu, {\sigma}^2\right)$ with expected value *μ* and variance *σ*^2^ at each stage. Then, we calculate the average KL divergence of the distribution of adjacent stages. If the average KL divergence of a feature is large, it more likely is a biomarker to distinguish different stages. The basic KL divergence of distribution *p*(*X*) and *q*(*X*) and the KL divergence of two univariate Gaussian distributions are in Eq. 10 and 11.

10 $$KL\Big(p(X)\mid \left|q(X)\right)=\sum_{x_i\epsilon X}p\left({x}_i\right) {\log}\frac{p\left({x}_i\right)}{q\left({x}_i\right)}$$

11 $$KL\left(\mathcal{N}\left({\mu}_1,{\sigma}_1^2\right)\Big\Vert \mathcal{N}\left({\mu}_2,{\sigma}_2^2\right)\right)=\log \frac{\sigma_2}{\sigma_1}+\frac{\sigma_1^2+{\left({\mu}_1-{\mu}_2\right)}^2}{2{\sigma}_2^2}-\frac{1}{2}$$

For measure and evaluate each feature, we use the average KL divergence (Average KL) between neighbor stages *g*_*i*_, *g*_*i* + 1_. *m* is the number of groups.

12 $$Average\ KL=\frac{1}{m}\sum_{i=0}^{m-1}{KL}_{g_i,{g}_{i+1}}$$

## Results

We used the records of 375 patients as a training set; the ratio of the training set to the verification set was 0.8:0.2. The records of 110 patients made up the test set. This experiment was conducted on 5-fold cross-validation. The code implementation is publicly available at https://github.com/scxhhh/COVID-19.

### Baselines

We use the related works summarized in Table 1 as comparison methods. Related works are divided into non-deep learning methods and deep learning methods. We use Cox [19], k-NN [16], SVM [17], DT [1], BPNN [20], PNN [21], RNN, LSTM and T-LSTM for COVID-19 mortality prediction. T-LSTM is our method.

### Outcome prediction results

Table 3 shows the results of COVID-19 mortality prediction using baselines. The AUC-ROC is evaluated at 0, 3, 6, 9, 12, 15, and 18 days early. Here, the results are obtained when the patient’s representations are 64 dimensional. The results indicate that our method T-LSTM performed better than all of baselines no matter how early before the onset times of patients. More precisely, using T-LSTM, the outcome prediction accuracy is above 90% at 12 days early and is approximately 97% accurate when predicting 3 days before the disease outcome. More detailed results of train, validation and test sets using T-LSTM are listed in Table 4.

**Table 3:** AUC-ROC of COVID-19 mortality prediction results by using baselines

	0 days early^a^	3 days early^a^	6 days early	9 days early	12 days early
Cox^b^	0.955 ± 0.06	0.992 ± 0.02	0.870 ±0.01	0.85 ±0.01	0.810 ±0.01
k-NN^c^	0.950 ± 0.02	0.909 ± 0.01	0.890 ±0.02	0.840 ±0.02	0.816 ±0.01
SVM^d^	0.969 ± 0.04	0.954 ± 0.02	0.930 ± 0.03	0.895 ±0.04	0.857 ±0.02
DT^e^	0.974 ±0.01	0.959 ± 0.03	0.924 ±0.00	0.897 ±0.01	0.869 ±0.03
BPNN^f^	0.980 ±0.02	0.954 ±0.05	0.933 ±0.0 1	0.894 ±0.02	0.878 ±0.03
PNN^g^	0.985 ±0.01	0.961 ±0.02	0.940 ±0.0 2	0.889 ±0.02	0.889 ±0.02
RNN^h^	0.985 ±0.01	0.960 ±0.01	0.931 ±0.00	0.910 ±0.02	0.871 ±0.01
LSTM^i^	0.990 ±0.01	0.961 ±0.02	0.937 ±0.02	0.920 ±0.03	0.897 ±0.03
T-LSTM^j^	**0.997** ***±*** **0.00**	**0.969** ***±*** **0.01**	**0.947** ***±*0.03**	**0.921** ***±*0.03**	**0.914** ***±*0.02**

^a^ n days early: The models make prediction n days before the final death/survival time. They use sequence data from day 0 to n days before the last time to predict

^b^ Cox: Cox’s proportional hazards regression model is semi parametric regression model. It can analyze the influence of many factors on outcomes. It is used in [19]

^c^ k-NN: k-Nearest Neighbors method makes prediction based on the information of nearest k samples in training set. In this mortality prediction task, the most accurate results appeared when k = 3

^d^ SVM: Support Vector Machines classify by solving the separation hyperplane which can divide the training data correctly and has the largest geometric interval

^e^ DT: Decision tree is a simple classifier consisting of sequences of hierarchically organized binary decisions. It is used in [33]

^f^ BPNN: Back Propagation Neuron Network makes the signal and the error propagate forward and backward separately. It is used in [20]

^g^ PNN: Probabilistic Neural Network is a forward propagation network and does not need back propagation to optimize parameters by using Bayesian decision-making. It is used in [21]

^h^ RNN: Recurrent Neural Network have been introduced in the ‘T-LSTM’ section

^i^ LSTM: Long Short-Term Memory which we have introduced in the ‘T-LSTM’ section. Here, the hyperparameter setting is same as T-LSTM

^j^ T-LSTM: Time-aware LSTM is the model used in this paper. Its inputs are the three-dimensional vectors and the time intervals. The values for each dimension are the values of LDH, lymphocyte and hs-CRP in patients’ blood tests. Its output is the binary result 0/1. Here, 0 indicates survival and 1 indicates death. The hidden states in its units are 64 dimensional, and the fully connected layer has 32 dimensions

**Table 4:** AUC-ROC of COVID-19 mortality prediction results by using T-LSTM on different sets at different timestamps

	Training^b^	Validation^b^	Test^b^
0 days early^a^	0.996 ± 0.01	0.997 ± 0.01	0.997 ± 0.00
3 days early	0.989 ± 0.00	0.987 ± 0.01	0.969 ± 0.01
6 days early	0.960 ±0.01	0.957 ±0.0 2	0.947 ±0.03
9 days early	0.944 ±0.00	0.935 ± 0.01	0.921 ±0.03
12 days early	0.926 ± 0.01	0.924 ±0.0 2	0.914 ±0.02
15 days early	0.891 ± 0.01	0.883 ± 0.01	0.863 ±0.01
18 days early	0.852 ± 0.01	0.834 ±0.0 2	0.819 ±0.0 2

^a^n days early: The model makes a prediction n days before the final death/survival time. It uses sequence data from day 0 to n days before the last time to predict

^b^We use the records of 375 patients as the training set; the ratio of training set to verification set is 0.8:0.2. The records of 110 patients make up the test set. This experiment is conducted on 5-fold cross-validation

The first four figures in Fig. 3 are the visualizes of prediction results. The first two figures are the AUC-ROC of prediction results of baselines and T-LSTM in different earliness. The third figure is the changes of prediction accuracy and cross-entropy loss when training the model. The fourth figure represents the relation of patient representation dimension and AUC-ROC of prediction using T-LSTM. Too few dimensions lead to incomplete feature learning, while too many dimensions lead to redundant calculations and easy over-fitting. Considering result accuracy, computational complexity and ease of representation use in the following task, we decided to use 64 dimensional vectors to represent patients.

Based on prediction results, we found: 1) Deep learning approaches (T-LSTM, RNN, PNN and BPNN) has higher COVID-19 outcome prediction accuracy than non-deep learning approaches (Cox, k-NN, SVM and DT) as they have completed the highly nonlinear feature transformation by neural junction structures. 2) RNN-based models (T-LSTM and RNN) performance better on time series data as they contain state connections for reproducing time delays and output feedback connections for forming a loop. 3) Time-aware model (T-LSTM) has the best performance as it can model the time series with irregular time intervals, which is a prominent feature of COVID-19 blood sample dataset.

Further, we also select 40 features (listed in Table 7) as the input of T-LSTM for comparative experiment. The results in Table 5 indicate that learning a large number of patient characteristics does not necessarily contribute to COVID-19 patient mortality prediction task. The three biomarkers, LDH, lymph and hs-CRP can make the results better. The AUC-ROC of using 3 features is 3% higher than using 40 features on average. This is because too many features will introduce redundant and irrelevant dependencies leading by redundant features.

**Table 5:** AUC-ROC of COVID-19 mortality prediction results by using T-LSTM with 40 or 3 laboratory tests^b^

	0 days early^a^	3 days early^a^	6 days early	9 days early	12 days early
40 features	0.949 ± 0.01	0.920 ± 0.03	0.915 ± 0.01	0.910 ± 0.01	0.903 ± 0.01
3 features	**0.997** ***±*** **0.00**	**0.969** ***±*** **0.01**	**0.947** ***±*0.03**	**0.921** ***±*0.03**	**0.914** ***±*0.02**

^a^ n days early: The model makes prediction n days before the final death/survival time. It uses sequence data from day 0 to n days before the last time to predict

^b^ The inputs of T-LSTM are time series of 48 laboratory tests or 3 laboratory tests (LDH, lymph and hs-CRP)

### Disease progression results

**Fig. 4:**
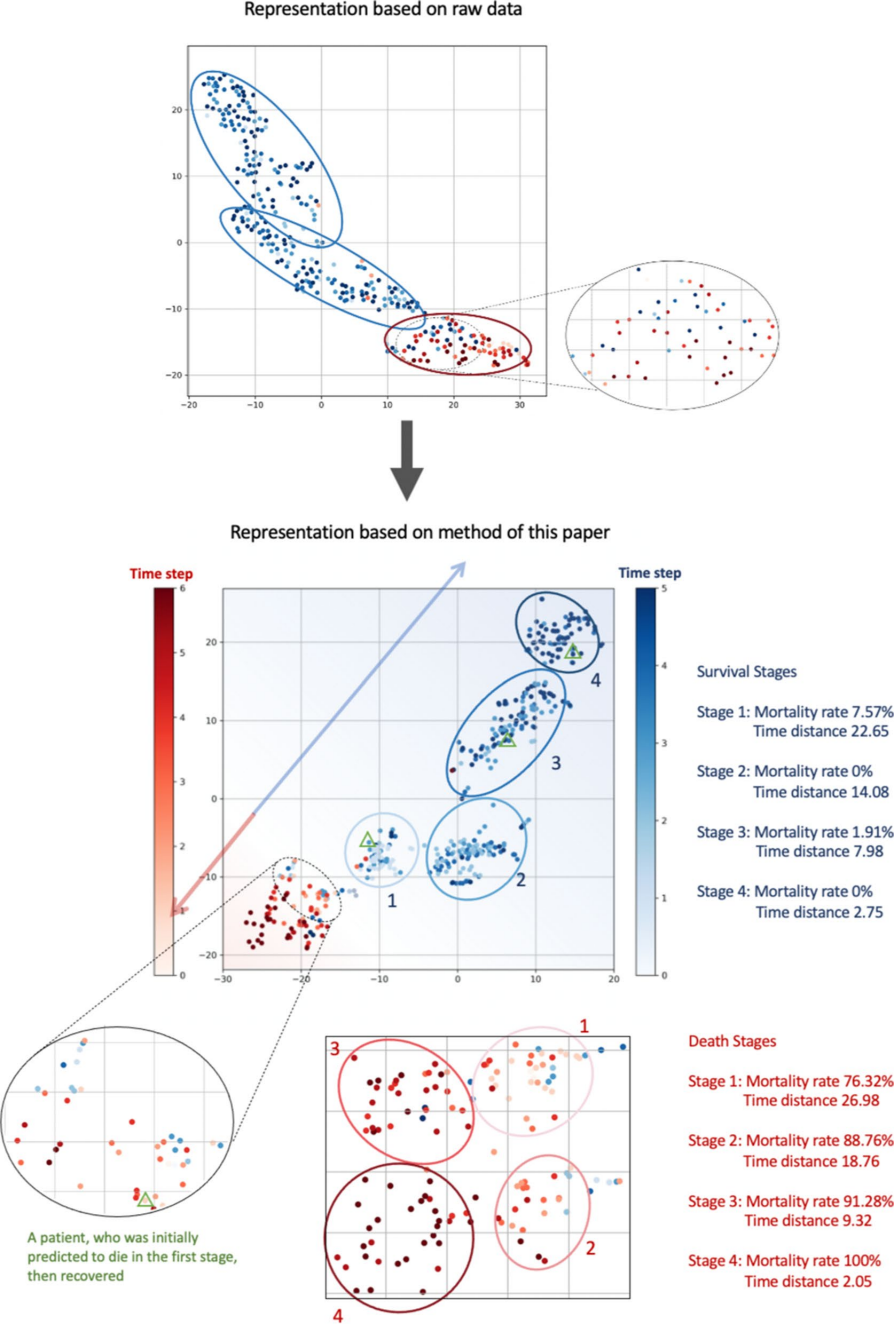
The result of COVID-19 progression. This figure shows the four stages of COVID-19 patients by using T-LSTM. The upper clusters are the original clustering of data. The lower are the patient subtyping by using T-LSTM. We can find there are four clusters with distinct boundaries both in death/critical class (red) and survival/general class (blue)

By implementing the four steps of disease progression mining, we obtained the 4 stages in both the death class (critical) and the survival class (general), shown in Fig. 4.

For better visualization, we reduced the dimension of the patient’s representation vector, the fifth figure in Fig. 3 is the dimension reduction effect. We chose 2 dimensions due to low representation loss and clear observation. Besides, the DBSCAN clustering effect evaluated by the CH index is shown in the sixth and seventh figures in Fig. 3. Different clustering effects can be obtained by changing the cluster radius parameter *ε*. The best CH index values for the death class and the survival class are 680.07 and 44.24, respectively.

In this case, both classes have four groups. Four stages of COVID-19 patients are shown in Fig. 4. For each stage, we calculate the mortality rate *MR* and the average time distance *TD*. For the death class, *MR* increases over stages and is 100% at stage 4. For the survival class, *MR* decreases over stages and is 0% in stage 4. *TD* in both classes decreases, meaning that the 4 stages are distributed over time. Meanwhile, as the CH index of the survival class is higher than that of the death class, the survival class stages are relatively loosely distributed.

In Fig. 4, the first clustering is obtained by using biomarkers directly and shows that reasonable stages could not be found. In the first clustering, no stage is clustered in the death class and the 2 stages in the survival class have similar mortality rates and no time difference, as the shade of blue indicates. However, using our method, different stages have obvious differences, such as the data point color deepening with the stages. Meanwhile, as shown in the two insets, the class boundary is clearer based on our method.

The division of stages contains the potential characteristics of COVID-19. Here, we present three findings. First, at the time of initial diagnosis, the COVID-19 infected patients’ physical conditions are similar, regardless of final survival or death. In Fig. 4, the distance between stage 1 for the death class and the survival class is small, and the two even overlap. This indicates that outcome prediction is likely not accurate at the time of infection. Second, the physical condition of patients who eventually die changes less than that of those who eventually survive. We conclude this from CH index values, where the CH value of the survival class is larger than that for the death class. Third, mortality rate varies by stage. For example, if the patient is classified into the death class and is at stage 1, there is still hope of survival, as shown by the green triangle sample in Fig. 4. However, if the patient is in stage 3 or 4, he or she is very likely to die. Based on estimating the current stage of a patient, doctors will be given a reference, which can help them assess a patient’s current situation. Based on that, doctors can carry out targeted treatment and reasonable resource allocation more easily. Thus, the ultimate goal of this study, helping improve the quality of medical care, can be achieved.

**Fig. 5:**
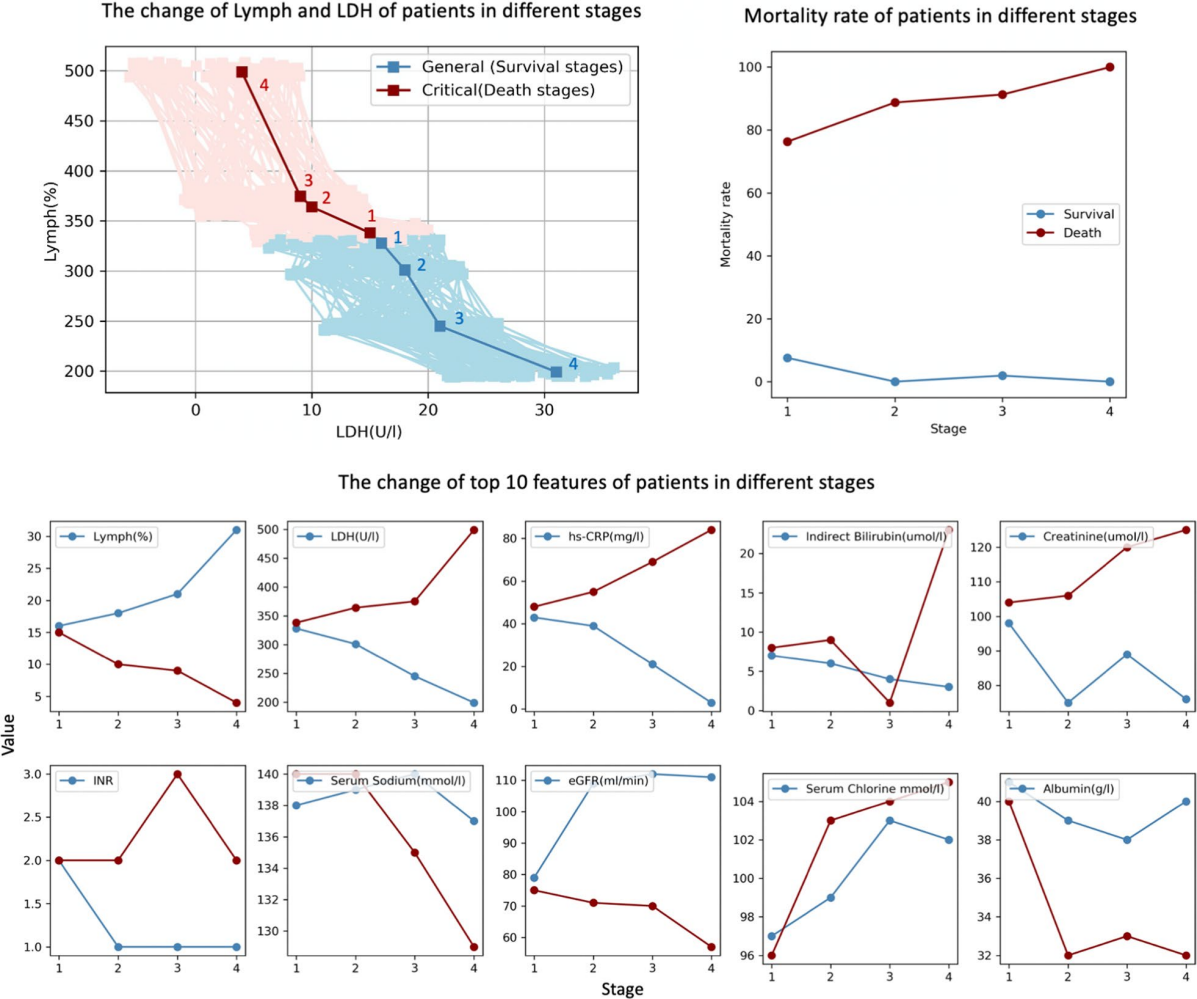
Changes of features in different stages. This figure shows the changes of features (Mortality rate, Lymph, LDH, hs-CRP, Indirect Bilirubin, Creatinine, INR, Serum Sodium, eGFR, Serum Chlorine and Albumin) through 4 stages. Under different classes, the trends of features are different

Meanwhile, we calculated the mean values of 40 laboratory test features in each stage, the feature values vary with stages. Table 6 lists 10 of these features - Lymph, LDH, hs-CRP, Indirect Bilirubin, Creatinine, INR, Serum Sodium, eGFR, Serum Chlorine and Albumin. The changes of values through 4 stages are visualized in Fig. 5. Under different classes, the trends of features are different.

**Table 6:** Feature statistics of patients in different stages of COVID-19 disease progression

Survival class (general)				
Mean value	Stage 1	Stage 2	Stage 3	Stage 4
Mortality rate (%)	7.57	0	1.91	0
Time distance (days)	22.65	14.08	7.98	2.75
Lymph (%)	16	18	21	31
LDH (U/l)	328	301	245	199
hs-CRP (mg/l)	43	39	21	3
Indirect Bilirubin (μmol/l)	7	6	4	3
Creatinine (μmoI/l)	98	75	89	76
INR	2	1	1	1
Serum Sodium (mmol/l)	138	139	140	137
eGFR (ml/min)	79	109	112	111
Serum Chlorine (mmol/l)	97	99	103	102
Albumin (g/l)	41	39	38	40
Death class (critical)				
Mean value	Stage 1	Stage 2	Stage 3	Stage 4
Mortality rate (%)	76.32	88.76	91.28	100
Time distance (days)	26.96	18.76	9.32	2.05
Lymph (%)	15	10	9	4
LDH (U/l)	338	364	375	499
hs-CRP (mg/l)	48	55	69	84
Indirect Bilirubin (μmol/l)	8	9	14	23
Creatinine (μmoI/l)	104	106	120	125
INR	2	2	3	2
Serum Sodium (mmol/l)	140	140	135	129
eGFR (ml/min)	75	71	70	57
Serum Chloride (mmol/l)	96	103	104	105
Albumin (g/l)	40	32	33	32

Further, we calculated the average KL divergence between adjoint stages of each features in 40 clinical laboratory tests data. We ranked the average KL values. The higher the ranking, the better the biomarkers can be used to distinguish different stages. By ranking 40 biomarkers according to the degree of correlation with COVID-19 (Table 7), we have found the biomarkers which are more relevant to COVID-19. The top 10 are: Lymph, LDH, hs-CRP, Indirect Bilirubin, Creatinine, INR, Serum Sodium, eGFR, Serum Chlorine and Albumin. For each marker, we gave its reference value in each stage, shown in Table 6. Different markers have unique trends in different stages.

**Table 7:** Ranking of average KL divergence values of top 40 features

Ranking	Feature	Average KL	Ranking	Feature	Average KL
1	Lymph	0.0421	21	Total Cholesterol	0.0041
2	LDH	0.0392	22	Interleukin 6	0.0032
3	hs-CRP	0.0376	23	I sIL-2R	0.0031
4	Indirect Bilirubin	0.0324	24	cTnI	0.0030
5	Creatinine	0.0302	25	RBCW-SD	0.0024
6	INR	0.0235	26	Uric Acid	0.0023
7	Serum Sodium	0.0232	27	Corrected Calcium	0.0022
8	eGFR	0.0225	28	Interleukin 8	0.0019
9	Serum Chloride	0.0224	29	Prothrombin Time	0.0018
10	Albumin	0.0193	30	Serum Potassium	0.0017
11	Globulin	0.0177	31	Interleukin 1β	0.0017
12	Hematocrit	0.0122	32	D-D dimer	0.0016
13	Hemoglobin	0.0091	33	FDP	0.0016
14	Fibrinogen	0.0079	34	Antithrombin	0.0015
15	γ-GT	0.0079	35	Procalcitonin	0.0010
16	ESR	0.0078	36	Platelet Count	0.0009
17	NT-proBNP	0.0074	37	WBC	0.0006
18	APTT	0.0053	38	Ferritin	0.0005
19	Eosinophils	0.0051	39	Interleukin 10	0.0004
20	basophil	0.0049	40	PLT	0.0004

Combining the correlation analysis with the reference value analysis, we found that the critical COVID-19 patients are usually accompanied by low values of lymph, eGFR, albumin and Serum Sodium, high values of LDH, hs-CRP, indirect bilirubin, creatinine and INR. For example, in the critical stage 4, the average lymph (%) is just 4 and the average LDH (U/l) is up to 499. Besides, there are three major complications of COVID-19 patients - myocardial injury, liver function injury and renal function injury. We got the conclusions separately through the value of 1) LDH, 2) albumin and indirect bilirubin, 3) serum sodium, serum chlorine and creatinine in different stages.

## Discussion

In recent years, deep learning (DL) technology has been widely used because of its superior performance in various medical applications [28, 29], such as medical image recognition [39] and medication recommendations [40]. In the past year, the spread of COVID-19 has had a peripheral effect on the global economy and health. Therefore, we expect to combine DL methods to study and fight COVID-19.

The states of COVID-19 patients in hospital are dynamic time sequence processes. In addition to the basic information of patients, the vital signs, diagnoses and other lab tests are all time series. Existing many works [14–27, 41, 42] have achieved good results for COVID-19 prediction tasks. But they paid little attention to analyze and model the characteristics of COVID-19 patients’ time series. Dynamic time series modeling can grasp the relationship between historical observations and current observations, and learn the potential development mode of sequence, which is conducive to more accurate prediction and representation. Besides, we have found that the time series of COVID-19 patients is irregularly sampled - Different time intervals exist in adjacent observations. Every possible test is not regularly measured during an admission. When a certain symptom worsens, corresponding variables are examined more frequently; when the symptom disappears, the corresponding variables are no longer examined. These time intervals will add a time sparsity factor when the intervals between observations are large [13]. Therefore, it is necessary not only to deal with time series, but also to deal with irregular time series according to the characteristics of COVID-19 patients. In this paper, we use time-aware LSTM model solved this problem.

Deep learning methods have outstanding performance in prediction tasks. If a doctor predicts survival or death only by observing the biomarkers and using a threshold, the accuracy is at or below 80% for early predictions. However, the clinical reference value of inaccurate results is very low [43, 44]. The DL method has better performance, and the time-aware aspect enables higher accuracy, as shown in Table 3.

However, there are some concerns about the use of DL methods in the high-risk tasks of healthcare.

First, it may be risky to apply predictive methods directly to clinical practice [45]. DL methods may be assistive tools for doctors but not used to make decisions directly. It is challenging for doctors to make optimal decisions, a data-driven and high-accuracy prediction method could help. In this paper, we can predict patient outcomes with higher accuracy than baselines. The method can effectively predict whether the infected patient will die or survive 12 days prior to disease outcome with over 90% accuracy. The prediction accuracies at 3-, 6-, and 9-days prior are 98, 95 and 93%, respectively.

Second, the DL method is the black-box models which are troubled by poor interpretability [46, 47], but clinical settings prefer interpretable models. For example, finding the appropriate prediction-related biomarkers is important. Currently, certain studies have identified suitable predictive biomarkers, such as the 3 biomarkers in [33], which are regarded to have a significant impact on patient mortality. For interpretability, our method identified four disease stages distributed over time. This interesting finding cannot be distinguished simply by the value of biomarkers, as shown as the comparison of two clustering results in Fig. 4. The discovered stages are closely related to mortality and time of illness and can help analyze the status of infected patients. This shows that the DL method can explore new patterns in multidimensional space that cannot be demonstrated by a simple variable value [48]. We also ranked 40 biomarkers according to the degree of correlation with COVID-19 progression, which can provide interpretable results to help doctors better understand the model.

This study has three basic contributions. 1) we can predict patient outcomes with higher accuracy than all baselines. 2) We identified four stages of COVID-19 progression. The stages are closely related to mortality and time of illness and can help analyze the status of infected patients. 3) We give the ranking of 40 biomarkers according to the degree of correlation with COVID-19. Based on this, we found three major complications of COVID-19 patients - myocardial injury, liver function injury and renal function injury.

Further, there is room for further improvement. First, because of the data limitations, our method may face risk of bias, because data-driven methods are easily influenced by different source of data. For example, the results may vary when using different datasets [45]. Second, our current interpretation is based on results, such as the degree of association between biomarkers and disease. We hope to give more explanations about the complex DL black-box model, such as telling more specific effect of each part of the model on the result. Meanwhile, we hope to enlighten the relevant researchers to further study these 4 stages and present more clinical explanations. In particular, we expect to be able to give specific treatments for different stages. Targeted treatment is significant for both patient rehabilitation and the reasonable allocation of medical resources.

## Conclusions

The sudden outbreak and epidemic of COVID-19 has led to worldwide suffering and shortages of medical resources. In this paper, we propose T-LSTM to predict patient outcomes with high accuracy - 98, 95 and 93% at 3, 6, and 9 days, which will enable reasonable allocation of medical resources. T-LSTM can effectively model the irregular sampled time series in blood test samples of COVID-19 patients and predict more accurately than existing baselines. Meanwhile, we identified four COVID-19 stages. We ranked 40 biomarkers according to correlations to the outcomes of patients, gave the reference values of top 10 biomarkers for each stage. The top 10 biomarkers are: Lymph, LDH, hs-CRP, Indirect Bilirubin, Creatinine, INR, Serum Sodium, eGFR, Serum Chlorine and Albumin. We also found 3 complications of COVID-19, which are myocardial injury, liver function injury and renal function injury. By analyzing patients’ life conditions at different stages, doctors can choose specific, targeted treatments. Future work will focus more on the study of pathological characteristics of different stages. Aiming at four stages, targeted treatments are expected to be designed. Meanwhile, more real clinical data are expected to be available for model validation and the model will be used to mine the inherent hidden features of other diseases.

## Data Availability

The code implementation is publicly available at https://github.com/scxhhh/COVID-19. The data is from an online open dataset https://github.com/HAIRLAB/Pre_Surv_COVID_19 under an MIT license (10.5281/zenodo.3758806).

## Abbreviations

COVID-19: Corona Virus Disease 2019
WHO: World Health Organization
LDH: Lactic Dehydrogenase
hs-CRP: High-sensitivity C-reactive Protein
lymph: Lymphocytes
DL: Deep Learning
RNN: Recurrent Neural Network
LSTM: Long Short-Term Unit
T-LSTM: Time-aware Long Short-Term Memory
PNN: Probabilistic Neural Network
RBFNN: Radial Basis Function Neural Network
GRNN: Generalized Regression Neural Network
BPNN: Back Propagation Neuron Network
DT: Decision Tree
RF: Random Forest
XGBoost: eXtreme Gradient Boosting
SVM: Support Vector Machines
Cox: Cox’s Proportional Hazards Regression
LR: Linear Regression
NB: Naive Bayes
LDA: Linear Discriminant Analysis
t-SNE: t-distributed Stochastic Neighbor Embedding
DBSCAN: Density-Based Spatial Clustering of Applications with Noise
MR: Mortality Rate
TD: Average Time Distance
AUC-ROC: The Area Under the Curve of the Receiver Operating Characteristic
CH: Calinski-Harabaz Index
KL divergence: Kullback-Leibler Divergence
